# Optimization of Point-Shear Wave Elastography by Skin-to-Liver Distance to Assess Liver Fibrosis in Patients Undergoing Bariatric Surgery

**DOI:** 10.3390/diagnostics10100795

**Published:** 2020-10-07

**Authors:** Mauro Giuffrè, Michela Giuricin, Deborah Bonazza, Natalia Rosso, Pablo José Giraudi, Flora Masutti, Stefano Palmucci, Antonio Basile, Fabrizio Zanconati, Nicolò de Manzini, Claudio Tiribelli, Silvia Palmisano, Lory Saveria Crocè

**Affiliations:** 1 Department of Medical, Surgical and Health Sciences, University of Trieste, 34149 Trieste, Italy; deborah.bonazza@asugi.sanita.fvg.it (D.B.); fabrizio.zanconati@asugi.sanita.fvg.it (F.Z.); ndemanzini@units.it (N.d.M.); spalmisano@units.it (S.P.); lcroce@units.it (L.S.C.); 2 Italian Liver Foundation, 34149 Trieste, Italy; natarosso@hotmail.com (N.R.); pablo.giraudi@fegato.it (P.J.G.); ctliver@fegato.it (C.T.); 3 General Surgery Clinic, Azienda Sanitaria Universitaria Giuliano-Isontina, 34149 Trieste, Italy; michela.giuricin@asugi.sanita.fvg.it; 4 Department of Pathology, Azienda Sanitaria Universitaria Giuliano-Isontina, Cattinara Hospital, 34149 Trieste, Italy; 5 Liver Clinic, Azienda Sanitaria Universitaria Giuliano-Isontina, Cattinara Hospital, 34149 Trieste, Italy; flora.masutti@asuits.sanita.fvg.it; 6 Department of Medical Surgical Sciences and Advanced Technologies G.F. Ingrassia University of Catania, 95124 Catania, Italy; spalmucci@unict.it (S.P.); basile.antonello73@gmail.com (A.B.)

**Keywords:** liver elastography, liver fibrosis, bariatric surgery, non-alcoholic fatty liver disease, abdominal wall thickness, abdominal wall, NAFLD, liver biopsy, liver histology, non-alcoholic steatohepatitis

## Abstract

Background: Obesity is a primary limiting factor in liver stiffness measurement (LSM). The impact of obesity has always been evaluated in terms of body mass index (BMI), without studying the effects of skin-to-liver distance (SLD) on LSM. We studied the impact of SLD on LSM in a cohort of obese patients undergoing bariatric surgery and intra-operatory liver biopsy. Materials and Methods: 299 patients underwent LSM by point-shear wave elastography (ElastPQ protocol), with two different ultrasound machines. SLD was measured as the distance between the skin and the liver capsule, perpendicular to where the region of interest (ROI) was positioned. We used the following arbitrary cut-offs: <5.7 kPa, F0–1; 5.7–7.99 kPa, F2; ≥8 kPa, F3–4. Results: We developed two logistic regression models using elastography–histology agreement (EHA) as the dependent variable and SLD as the independent variable. The model based on the second machine showed strongly more performant discriminative and calibration metrics (AIC 38.5, BIC 44.2, Nagelkerke Pseudo-R2 0.894, AUROC 0.90). The SLD cut-off value of 34.5 mm allowed a correct EHA with a sensitivity of 100%, a specificity of 93%, negative predictive value of 100%, positive predictive value of 87%, an accuracy of 96%, and positive likelihood ratio of 3.56. Conclusion: The impact of SLD is machine-dependent and should be taken into consideration when interpreting LSM. We believe that our findings may serve as a reference point for appropriate fibrosis stratification by liver elastography in obese patients.

## 1. Introduction

Obesity is a global epidemic, with an estimated number of 650 million obese adult individuals worldwide [1]. Obesity is directly related to the development of non-alcoholic fatty liver disease (NAFLD) [2]. NAFLD is characterized by a disease spectrum ranging from simple hepatic steatosis (i.e., non-alcoholic fatty liver, NAFL) to non-alcoholic steatohepatitis (NASH) in as many as 40% of patients [3,4,5]. Over time, NASH can further promote liver injury, causing fibrosis and, potentially, cirrhosis [3]. Therefore, appropriate fibrosis staging and monitoring are crucial in obese individuals [6].

Over the last decade, elastography has attracted a great deal of interest for non-invasive measurement of tissue elasticity [7] overcoming other non-invasive fibrosis scores [8]. Unfortunately, obesity is a primary limiting factor to elastography accuracy [9]: so much that it has driven transient elastography (TE) producers to develop probes with augmented penetrance [10]. However, the success rate is technique-dependent: a recent meta-analysis demonstrated that TE has a higher failure rate in obese patients if compared to point-Shear Wave Elastography (pSWE) (11.3% vs. 0.8%) [11]. Despite the higher success rate of pSWE, few studies have addressed the lack of cut-off values to discriminate between liver fibrosis stages [12]. In addition, there is a widespread paucity of data about the effect of skin-to-liver distance (SLD) on liver elastography feasibility and accuracy. Preliminary results showed that a higher SLD reduces the confidence in Liver Stiffness Measurement (LSM) [13,14] and increases the rate of unreliable LSM results. In particular, LSM could optimally discriminate between fibrosis stages for SLD <34 mm [15].

Therefore, the present study aimed at establishing the effect of SLD on LSM reliability in a cohort of obese patients with a biopsy-proven staging of liver fibrosis. Secondary aims included the investigation of the agreement between LSM performed by two different machines that employ the same elastography protocol. We also investigated the ability of the ultrasonographic Hamaguchi Score [16] to predict the histological degree of steatosis.

## 2. Material and Methods

We enrolled consecutive patients who were selected for elective bariatric surgery and referred to the Surgical Clinic Unit at Cattinara Hospital.

All procedures performed in studies involving human participants were conducted following the ethical standards of the institutional and/or national research committee and with the 1964 Helsinki declaration and its later amendments or comparable ethical standards. Comitato Etico Regionale Unico, FVG, SSN (Local Ethical Committee)—Protocol Number 22979 approved on 15 October 2015. Informed consent was obtained from all individual participants included in the study. Consenting patients were included according to international guidelines: well-informed and motivated patients with acceptable operative risks, failure of non-surgical treatments, declared compliance to follow lifelong medical surveillance, aged from 18 to 65 years, with a BMI of 40 kg/m^2^ or between 35 and 40 kg/m^2^ with obesity-related comorbidities [17]. Besides, to overcome the low-number of bariatric patients with higher degree of fibrosis, we prospectively enrolled patients evaluated by the Liver Center with anthropometric and metabolic characteristics compatible to the inclusion criteria for bariatric surgery, who had undergone liver biopsy as for the standard clinical practice and presented histological significant fibrosis and without clinical/ultrasonographic/endoscopic signs of hepatic decompensation. Baseline anthropometric and metabolic parameters were registered before the surgery, including height (cm), body weight (kg), sex, and BMI (kg/m^2^). Metabolic parameters included the following: glycated hemoglobin (HbA1c), fasting plasma glucose (mg/dL), fasting plasma insulin (mIU/L), HOMA Insulin-Resistance (HOMA-IR) Index, high-density lipoprotein cholesterol (HDL-C), low-density lipoprotein cholesterol (LDL-C), and triglycerides. Each patient underwent hepatotropic viruses (hepatitis A, hepatitis B, and C viruses) screening and liver function tests, including aspartate aminotransferase (AST, U/L), alanine aminotransferase (ALT, U/L), gamma-glutamyl transpeptidase (GGT, U/L), alkaline phosphatase (ALP, U/L), total bilirubin (mg/dL), and albumin (g/dL).

### 2.1. Liver Elastography

Liver stiffness measurement (LSM) was performed the day before bariatric surgery. LSM was performed with pSWE technology and ElastPQ evaluation protocol, using two different Philips (Philips Healthcare, Best, Netherlands) ultrasound systems: IU22 (machine 1) and Affiniti 70 (machine 2), with a 1–5 MHz convex probe. The exact acquisition of LSM, patients’ preparation and reliability criteria were already explained elsewhere [18,19,20]. Of notice, the region of interest (ROI) was always positioned at two centimeters from the liver capsule. Given the risk of overlap between LSM among fibrosis groups, we employed the following liver stiffness cut-offs derived from our previous experience in healthy individuals [21] and from literature [22,23] to stage liver fibrosis: <5.7 kPa, F0–1; 5.7–7.99 kPa, F2; >8 kPa F3–F4. In the first three years of the study, LSM was measured by four different operators with different degree of expertise (each of them had at least one year in ultrasound training and had performed at least one hundred liver elastographies). In the last three years of the study, LSM was measured alternatively by six operators with an expertise compatible to the previous operators. To study the inter-system agreement, seventy-five patients underwent liver elastography with both machines, in a double-blind fashion between two different operators.

SLD was considered as the distance between skin and Glissonian capsule, measured in millimeters (mm) with the probe positioned in the intercostal window. The SLD was measured perpendicularly to the superior border of the ROI for each valid elastographic measurement, and the median value was used for analysis.

### 2.2. Ultrasound Evaluation of Steatosis

After each elastography, the patients underwent ultrasonographic examination of the liver, gall bladder, spleen, and kidneys. To assess the severity of liver steatosis, we used the Hamaguchi Score (HS) [16], which evaluates hepatorenal echo contrast, bright liver, deep attenuation, and vessel blurring. Each item was acquired as explained by the authors [16].

### 2.3. Liver Biopsy

All surgical procedures were conducted laparoscopically. Before starting with the bariatric procedures, surgical liver biopsy was performed on the free margin of the left lobe. The specimen was approximately 1.5 × 2 cm large. In the operating room, the surgical biopsies were stored in formalin solution and sent for histopathological analysis. Only those samples more extended than 14 mm on histological examination were included in the study. The liver tissue, after formalin fixation, was stained with hematoxylin-eosin and with Masson’s Trichrome Staining. We used the scoring system developed by Kleiner et al. to stage fibrosis (0 = absence of fibrosis; 1a–b = perisinusoidal fibrosis; 1c = periportal or portal fibrosis; 2 = perisinusoidal and portal/periportal fibrosis; 3 = septal or bridging fibrosis; 4 = liver cirrhosis) and steatosis (grade 0 = steatosis <5%; grade 1 = steatosis 5–33%; grade 2 = steatosis ≥34–66%; grade 3 = steatosis >66%) [24].

### 2.4. Statistical Analysis

According to the size of our sample, the Shapiro–Wilk test was performed to verify the normal distribution of variables. Accordingly, variables were reported as median (quartile 1; quartile 3) or mean (± standard deviation). Prior to any further statistical analysis, we investigated anthropometrical and metabolic variations between patients with different degree of fibrosis (i.e., F0–1 vs. F2; F0–1 vs. F3–4, and F2 vs. F3–4) using the Mann–Whitney U or the Wilcoxon Sum-Rank tests for continuous variables, and the Pearson’s Chi-Square test for discrete variables. As expected, we did not detect any statistically significant inter-group difference. Correlations within variables were explored using the Spearman’s rank correlation coefficient. Choen’s kappa [25] was used to evaluate the agreement on fibrosis grading between elastography and biopsy. The discriminatory ability of LSM between fibrosis stage was initially explored thorough c-statistics, and inter-curve differences were investigated using the Hanley–McNeil method [26]. We defined the elastography–histology agreement (EHA) as the agreement between fibrosis staging with LSM using the cut-offs mentioned in the liver elastography section and the histological staging of liver fibrosis. Accordingly, the term “correctly classified” is referred to as the percentage of patients with EHA for each fibrosis stage.

The association (discrete; 0 = no, 1 = yes) between EHA and the SLD was evaluated using logistic regression models [27]. The independent variables were all modeled as continuous. To calculate the expected probability plot, each linear predictor (LP) was employed in the following function:

$$f\left(LP\right)=1-\left(\frac{1}{1+\;e^{LP}}\right)$$

We compared models using the Akaike information criterion (AIC) and the Bayesian information criterion (BIC) and calculated Nagelkerke pseudo-R2 and the area under the receiver-operating characteristic curve (AUROC) [28]. The discriminatory ability of HS was investigated through c-statistics, transforming Kleiner steatosis score into discrete variables (0 = steatosis <5%; 1 = steatosis ≥5%). The diagnostic accuracy of SLD and HS was calculated using sensitivity, specificity, positive predictive value (PPV), negative predictive value (NPV), accuracy, positive likelihood ratio (+LR), and negative likelihood ratio (−LR). Optimal cut-offs were chosen by selecting the value of SLD with highest sensitivity.

The sample size for the inter-machine agreement was calculated according to the method proposed by Liao [29]: with a tolerance probability (β) of 90% and a discordance rate (α) equals to 0.05, we must include at least 45 individuals. The inter-machine agreement was expressed in terms of the intra-class correlation coefficient (ICC) [30]. Bland–Altman plot [31] was employed to estimate the rate of systematic inter-machines over/under-estimation. For all analyses, two-sided statistical significance was defined as *p* < 0.05. Data were analyzed using SPSS (Statistical Package for Social Science) version 25.0 (IBM SPSS Statistics for MAC OS. Armonk, NY:IBM Corp.).

## 3. Results

From March 2014 to February 2020, 261 patients underwent bariatric surgery. During the same time-interval, only thirty-eight non-surgical patients (with compatible anthropometric and metabolic characteristics to the cohort of obese individuals) showed histological results compatible with significant liver fibrosis (Kleiner 3–4). As shown in Table 1, most of the patients were female (62.2%), with a median age of 53 (41; 57) years. One hundred and sixty (53.5%) patients had insulin-resistance, and 49 (16.4%) were diagnosed with T2DM. Although the median serum transaminase level was in the range of normality, 52 (17.4%) patients showed elevated transaminase levels. Of notice, none of the patients showed serum transaminase more than 2-times greater than the upper reference value. Regarding liver histology, the majority of the patients (65.9%) showed F0–1 score, and grade 0–1 steatosis in 60.2% of patients. A weak linear correlation was found between SLD and BMI (*r* = 0.441, *p* < 0.001), waist circumference (*r* = 0.295, *p* = 0.010), and HOMA-IR Index (*r* = 0.210, *p* = 0.022).

### 3.1. The Impact of SLD on Philips IU22 Measurements

**Figure 1:**
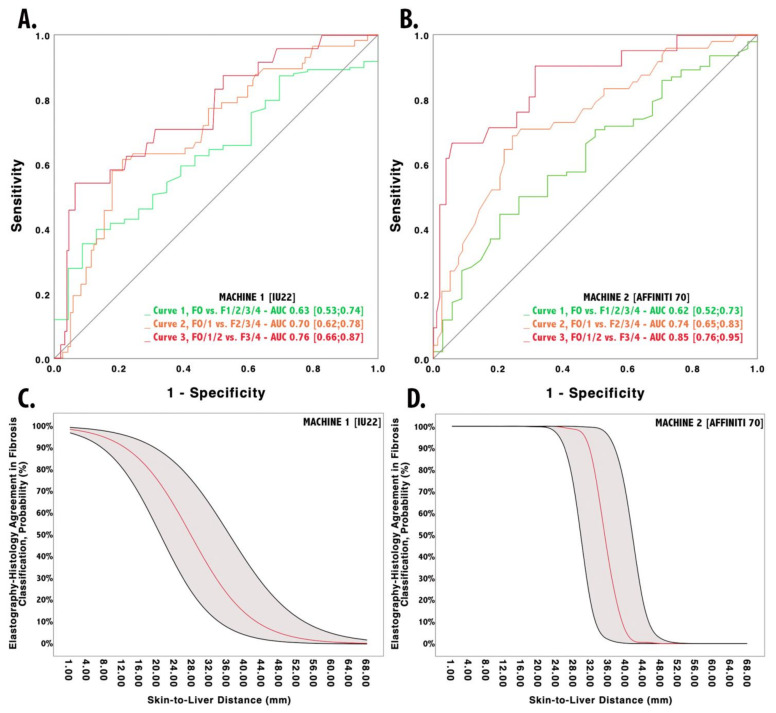
Discriminative ability of liver stiffness—measured by Philips IU22 (**A**) and Philips Affiniti 70 (**B**)—between various degrees of fibrosis. Probability of agreement between histology and elastography based on Skin-to-Liver Distance according to Philips IU22 (**C**) and Philips Affiniti 70 (**D**).

One hundred and ninety-three patients were evaluated by the Philips IU22 system. Ten patients (5.2%) were excluded because they had unreliable results. Patients’ LSM were stratified according to liver fibrosis staging on liver biopsy (Table 2). The median LSM for patients with F0, F1, and F2 stages was not statistically different. However, patients with fibrosis F3–4 had a statistically significantly higher median LSM if compared to patients without fibrosis F0 (*p* < 0.0001) or with fibrosis F1 (*p* < 0.0001) or F2 (*p* = 0.002). The discriminative ability of LSM was investigated through ROC analyses, as shown in Figure 1. The Hanley–McNeal method did not demonstrate any statistically significant inter-curve difference. Given the LSM cut-offs proposed in the methods section, 72 (39.3%) patients were correctly classified according to histological staging (Choen’s kappa = 0.376, *p* < 0.001). Patients with an EHA had a median SLD of 27 (23; 30) mm, which was significantly lower (*p* < 0.001) if compared to patients without EHA: median SLD of 34 (30; 39) mm.

Metrics of the logistic regression model to study the association of SLD and EHA, are reported in Table 3, whereas the derived probability is reported in Figure 1. According to the function, the EHA probability slowly diminishes with SLD increase. In particular, EHA probability is (1) greater than 80% in patients with lower SLD (<18 mm), (2) lower than 50% with SLD over 28 mm, and (3) less than 20% for thicker SLD (>37 mm). The SLD cut-off value that allowed us to rule out the presence of any false negative was 19 mm. This value showed a sensitivity of 100%, a specificity of 62%, NPV of 100%, PPV of 7%, an accuracy of 63%, and +LR of 2.7.

### 3.2. The Impact of SLD on Philips Affiniti 70 Measurements

One hundred and fifty-six patients were evaluated by Philips Affiniti 70 system. Eleven patients (7%) were excluded because they had unreliable results. Patients’ LSM were stratified according to liver fibrosis staging on liver biopsy (Table 2). The median LSM for patients with F0, F1, and F2 stages were not statistically different. However, patients with fibrosis F3–4 had a significantly higher median LSM if compared to patients without fibrosis F0 (*p* < 0.0001) or with fibrosis F1 (*p* < 0.0001) or F2 (*p* = 0.010). The discriminative ability of LSM was investigated through ROC analyses, as shown in Figure 1. The Hanley–McNeal method did demonstrate a statistically significant inter-curve difference between curve 1 and curve 3 (*p* = 0.002), whereas other inter-curve differences did not result statistically significant (curve 1 vs. curve 2, *p* = 0.08; curve 2 vs. curve 3, *p* = 0.10).

Given the LSM cut-offs proposed in the methods section, 76 (52.4%) patients were correctly classified according to histological grading (Choen’s kappa = 0.521, *p* < 0.0001). Patients with an EHA had a median SLD of 26 (23;30) mm, which was significantly lower (*p* < 0.0001) than in patients with discordant grading, who showed a median SLD of 43 (40;47.3) mm.

Metrics of the logistic regression model to study the association of SLD and EHA are reported in Table 3, whereas the derived probability is reported in Figure 1. According to the function, the EHA probability rapidly decreases with SLD greater than 30 mm. In particular, the EHA probability drops from 95% to less than 5% for SLD between 30 and 40 mm. The SLD cut-off value that allowed us to rule out the presence of any false negatives was 34.5 mm. This value showed a sensitivity of 100%, a specificity of 93%, NPV of 100%, PPV of 87%, an accuracy of 96%, and +LR of 3.56.

### 3.3. Inter-Machine Liver Stiffness Measurement Agreement

**Figure 2:**
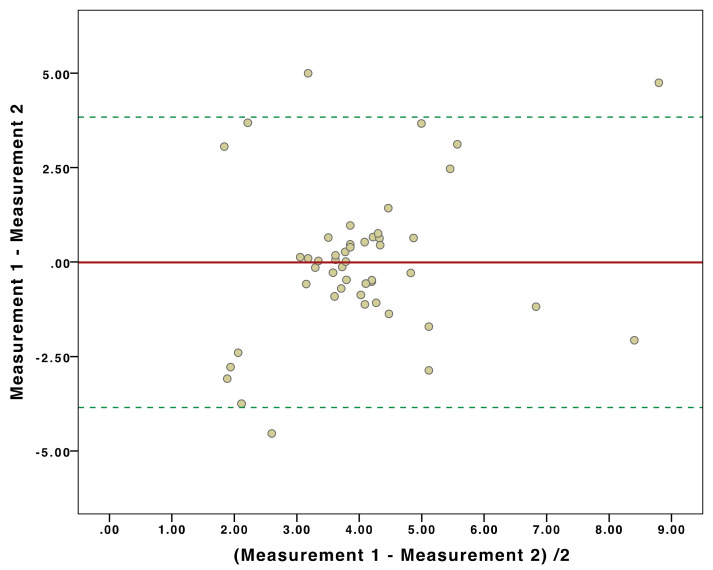
Bland–Altman plot regarding liver stiffness measurements by machine 1 and machine 2 on the same patients. The mean difference for liver stiffness was −0.0072, with 95% limits of agreement between −3.85 and 3.84. It shows no systematic overestimation or underestimation between machine 1 and machine 2.

Fifty patients with reliable results underwent LSM by Philips IU22 and Philips Affiniti 70. The ICC for single measures was 0.876 (*p* = 0.011) and 0.892 for average measures (*p* = 0.009). Bland–Altmann’s plot is represented in Figure 2, which added to the linear regression analysis between the difference and mean of each LSM by the two machines, did not result statistically significant (*p* = 0.97), thus, demonstrating no systematic overestimation or underestimation (i.e., proportional bias) between the two systems.

### 3.4. Application of Hamaguchi Score for Steatosis

**Figure 3:**
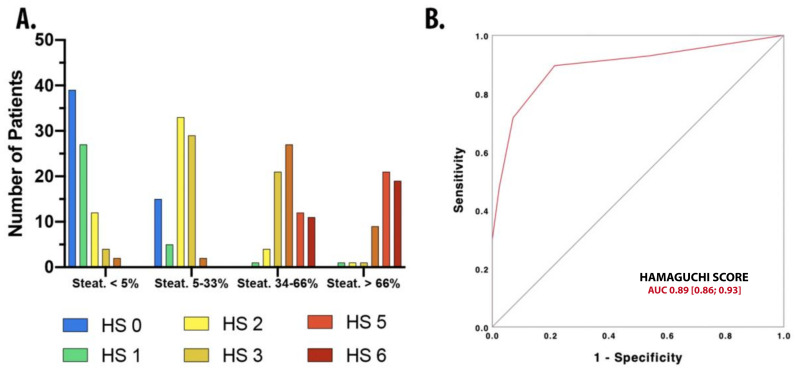
(**A**) Distribution of HS between Kleiner steatosis scores. (**B**) ROC employed to describe HS discriminative ability of HS for steatosis >5% as proven by liver histology. HS = Hamaguchi Score.

All patients enrolled in the study were clustered according to the histological degree of steatosis. The distribution of Hamaguchi scores across the various degree of steatosis is shown in Figure 3. The discriminative ability of HS was investigated through c-statistics, which demonstrated an AUROC of 0.89 (95% C.I., 0.86; 0.93). Using a HS ≥2, it was possible to discriminate steatosis with a sensitivity of 89.6%, a specificity of 78.6%, PPV of 91.4%, NPV of 75%, +LR of 4.18, –LR of 0.13, and accuracy of 86.5%.

## 4. Discussion

The main aim of liver elastography introduction in the daily clinical practice is to replace the need of liver biopsy, which despite its invasive nature [32], sampling errors [33], and intra/inter-observer variability [34], remains the gold standard in hepatic fibrosis staging [35]. In addition, liver elastography could be proposed as a non-invasive tool to stage fibrosis also in high-risk patients who would not have indications for liver biopsy, such as obese patients.

Given the risks of underestimation of liver fibrosis, it is crucial to interpret elastography results in the light of possible confounding factors that may influence LSM (such as liver inflammation, hepatic congestion from right heart failure, alcohol consumption, cholestasis, and fasting state) [36]. As reported in Table 1 and in the methods section, patients with such confounding factors were excluded, with the only exception of aminotransferases moderate increase (up to 2-times above the ULN) that was found in 52 (17.4%) patients, a level that seems not to affect LSM [37,38].

Despite the absence of confounding factors, LSM seemed to poorly discriminate between fibrosis stages, especially for patients with fibrosis < F3–4 stages, if SLD is not taken into account. These findings are in line with what has been reported by several studies, which demonstrated how LSM could not discriminate between lower fibrosis stages (F ≤ 2) [11], especially in patients with a BMI ≥ 30 kg/m^2^ [10]. Moreover, by using LSM cut-offs proposed in the methods section, only 72 (39.3%) patients were correctly classified with machine 1 (Choen’s kappa = 0.376, *p* < 0.0001) and 76 (52.4%) with machine 2 (Choen’s kappa = 0.521, *p* < 0.0001). Collectively, these findings led us to investigate the role of SLD as a confounding factor for effective LSM through logistic regression analysis. As interpreting logistic regression models may result complex, before reading the following part of discussions, check the plot in Figure 2 to visually evaluate the effect of SLD on LSM. The two models showed discriminative and calibration metrics with values strongly in favor for machine 2 (AIC 38.5, BIC 44.2, Nagelkerke Pseudo-R2 0.894, AUROC 0.90). In fact, pSWE can discriminate between F0–1, F2, and F3–4 stages if SLD is considered, whose role can be synthesized into two simple rules: (1) the thicker the abdominal wall, the lesser accuracy for a correct histological staging, and (2) the impact of SLD into correct fibrosis staging is machine-dependent. For example, machine 2 maintained a 95% probability of correct fibrosis staging for SLD equals to 30 mm, a thickness that could lead to a high risk of improper fibrosis staging with machine 1 (<40% probability of agreement). The most suitable SLD for appropriate fibrosis staging with machine 1 was 18 mm. Therefore, machine 2 kept higher accuracy even with a thicker abdominal wall, which may unsharp ultrasounds propagation and could lead to an altered analysis of returning waves [39]. We attributed this difference in performance to the fact that machine 2 is an upgraded version of machine 1, where the manufacturer had introduced substantial upgrade in ElastPQ algorithm, allowing for higher LSM performances even in more challenging patients, despite maintaining similar failure rates (5.2% vs. 7%) and excellent inter-machine ICC, without any systematic over/under-estimation of liver stiffness. However, it is important to emphasize that LSM were carried out by ten different operators over the course of six years. Despite the theoretical high variability that such a variegate pool of operators may have introduced to the results, in a previous study, we reported an inter-operator ICC of 0.93 evaluated through double-blind LSM. Therefore, it is highly unlikely that the number of operators may have affected the results of the current study.

Despite the mere staging of fibrosis, an accurate estimation of liver lipidic drops distribution is essential in the diagnostic work-up of patients with NAFLD because liver steatosis is linked to an increased prevalence of metabolic syndrome and cardiovascular risk. The Hamaguchi scoring system is characterized by excellent intra- and inter-observer reliability [16]. In addition, according to the current literature, HS ≥2 is highly specific and sensitive to diagnosing NAFLD [40], which was also confirmed in our patients (HS ≥ 2 showed a sensitivity 89.6% and specificity 78.6%).

One of the most consistent limitations of the study is related to the different location of liver biopsy (left lobe) and LSM (right lobe). However, in obese patients, histological findings between the two liver lobes showed an agreement of 93% for steatosis and 98% for fibrosis [41]. Besides, the distribution of the enrolled population was strongly skewed towards patients without higher stages of liver fibrosis, which is related to the peculiarities of liver disease in the bariatric population [42], which lead us to enroll an external cohort of patients with significant liver fibrosis. Despite these considerations, this is the first report of SLD as a pivotal confounding factor in liver elastography in such a numerous cohort of patients. Moreover, the novelty of the current study consists in the possibility of the proposition of reliable LSM cut-offs for appropriate fibrosis staging, taking into consideration that SLD may influence the reliability of LSM. We believe that our study may serve as a reference point for appropriate fibrosis stratification by liver elastography in obese patients.

## Figures and Tables

**Table 1:** Patients’ clinical, biochemical and histological characteristics. Continuous variables are reported as median (Quartile1; Quartile3) or mean (± SD). Discrete variables are reported as the number and proportion of subjects with the characteristic of interest. Abbreviations: BMI = body mass index; SLD = Skin-to-Liver Distance; HDL = high-density lipoprotein; LDL = low-density lipoprotein; AST = aspartate aminotransferase; ALT = alanine aminotransferase; GGT = gamma-glutamyl-transferase; ALP = alkaline phosphatase.

Characteristic	Value
Age, years	53 (41; 57)
Gender, female, *n* (%)	174 (62.2 %)
BMI, kg/m^2^	44 (40; 50)
Waist Circumference, cm	135 (121; 146)
SLD, mm	32 (26; 40)
Glycated Hemoglobin, %	6.3 (± 0.8)
Fasting Plasma Glucose, mg/dL	110 (99; 131)
Fasting Plasma Insulin, mIU/L	20 (13; 36)
HOMA-IR Index	5.10 (3.85; 8.53)
HOMA-IR Index >2.73, *n* (%)	160 (54.7%)
Previous Diagnosis of Type 2 Diabetes Mellitus, *n* (%)	49 (16.4%)
HDL Cholesterol, mg/dL	38 (36;43)
LDL Cholesterol, mg/dL	171 (151; 199)
Triglycerides, mg/dL	143 (110; 210)
AST, U/L	26 (21; 32)
ALT, U/L	29 (23; 39)
GGT, U/L	33 (21; 49)
ALP, U/L	75 (64; 102)
Total Bilirubin, mg/dL	0.5 (0.34; 0.68)
Albumin, g/dL	3.9 (3.7; 4.4)
Liver Fibrosis (Kleiner), *n* (%) Score 0 (F0) Score 1 (F1) Score 2 (F2) Score 3 (F3) Score 4 (F4)	55 (18.4%) 142 (47.5%) 59 (19.7%) 19 (6.4%) 24 (8%)
Liver Steatosis Grade, *n* (%) Score 0 Score 1 Score 2 Score 3	74 (24.8%) 106 (35.4%) 71 (23.8%) 48 (16%)

**Table 2:** Liver Stiffness measured by the two machines Philips IU 22 and Affiniti 70 and compared by histological grading of fibrosis. In patients evaluated by Philips IU 22, higher degree of fibrosis (F3–4 group) showed statistically significant higher liver stiffness if compared to F0 (*p* < 0.0001), F1 (*p* = 0.0001), and F2 (*p* = 0.002). In addition, in patients evaluated by Philips Affiniti 70, higher degree of fibrosis (F3–4 group) showed statistically significant higher liver stiffness if compared to F0 (*p* < 0.0001), F1 (*p* = 0.0001), and F2 (*p* = 0.010).

**Fibrosis Staging According to Philips IU 22 (*n* = 183)**				
Values	Kleiner F0, *n* = 24	Kleiner F1, *n* = 101	Kleiner F2, *n* = 33	Kleiner F3–4, *n* = 25
Liver Stiffness (kPa), Median (IQR)	4.1 (3.6;5.3)	4.34 (3.6;5.5)	6.6 (4;6.7)	8.1 (4.9;9.4)
Correctly Classified by Arbitrary Cut-Off	15/24 (62.5%)	25/101 (24.75%)	18/33 (54.5%)	14/25 (56%)
**Fibrosis Staging According to Philips Affiniti 70 (*n* = 125)**				
Values	Kleiner F0 *n* = 45	Kleiner F1 *n* = 54	Kleiner F2 *n* = 26	Kleiner F3–4 *n* = 20
Liver Stiffness (kPa), Median (IQR)	4.5 (3.9;6.20)	4.6 (4.1;6.10)	6.5 (4.3;7.2)	8.6 (6.3;9.3)
Correctly Classified by Arbitrary Cut-Off	29/45 (64.4%)	17/54 (31.5%)	14/26 (53.8%)	16/20 (80%)

**Table 3:** Logistic regression models metrics. Values are regression coefficients and robust 95% confidence intervals from logistic regression and model discriminative and calibration metrics.

Logistic Regression Probability Models		
Impact of Abdominal Wall Stiffness on Elastography–Histology Agreement		
Variables	[M1] Philips IU 22	[M2] Philips Affiniti 70
Abdominal Wall Thickness	−0.144 [−0.16;−0.13]	−0.591 [−0.617;−0.564]
Intercept	3.99 [3.35;3.63]	20.86 [18.3;23.4]
Nagelkerke Pseudo-R2	0.266	0.894
AIC	207.7	38.5
BIC	214.1	44.2
AUROC	0.79 [0.72;0.86]	0.90 [0.85;0.93]
Hosmer–Lemeshow *p*-value	0.09	0.15

## Abbreviations

NAFLD	non-alcoholic fatty liver disease
NAFL	non-alcoholic fatty liver
NASH	non-alcoholic steatohepatitis
LD	linear dichroism
TE	transient elastography
pSWE	point Shear Wave Elastography
AWT	abdominal wall thickness
LSM	liver stiffness measurement
ElastPQ	Elastography Point Quantification
HS	Hamaguchi Score
BMI	body mass index
EHA	elastography–histology agreement
ALT	alanine aminotransferase
AST	aspartate aminotransferase
GGT	gamma-glutamyl-transferase
HDL	high density lipoprotein
LDL	low density lipoprotein
IQR	interquartile range
